# Definition and conceptual framework of the psychological domain of intrinsic capacity in healthy ageing: outcomes from the MOSAIC International Working Group

**DOI:** 10.1016/j.jnha.2026.100969

**Published:** 2026-08-28

**Authors:** Neta Hagani, Alice Windle, Candice Oster, Sonia Hines, Jyoti Khadka, Vivian Isaac

**Affiliations:** a School of Allied Health, Exercise and Sports Sciences, Faculty of Science, Charles Sturt University, New South Wales, Australia; b College of Health and Enablement, Caring Futures Institute, Flinders University, South Australia. Sturt Rd, Bedford Park, South Australia, Australia; c College of Human Science and Culture, Open Door, Flinders University, South Australia. Sturt Rd, Bedford Park, South Australia, Australia; d School of Public Health, Adelaide University, South Australia, Australia

**Keywords:** Healthy ageing, Intrinsic capacity, Psychological capacity, Measurement development, Framework, MOSAIC

## Abstract

Healthy ageing relies on maintaining functional ability, shaped by both intrinsic capacity and environmental factors. Psychological capacity, a domain of intrinsic capacity, lacks clear conceptualisation and measurement for monitoring healthy ageing. To address this, an International Working Group held two virtual meetings in late 2025 with 24 experts from six World Health Organization (WHO) regions to develop a working definition and a conceptual framework for measuring psychological capacity in healthy ageing. Using a structured process, the group reviewed the potential attributes and criteria for measurement and proposed the following working definition: *Psychological capacity describes all the psychological attributes that contribute to an individual’s functional ability. It comprises intrinsic psychological characteristics, assets and skills that enable the individual to respond to internal stressors and contextual demands. These capacities are expressed as flexible attributes that enable cognitive appraisal, affect and emotional regulation, adaptability, coping, social competence, and personal agency.* The resulting definition and conceptual framework provide an important foundation for the measuring and monitoring of psychological capacity in healthy ageing.

## 1 Introduction

Demographic shifts driven by longer life expectancy and lower fertility rates have made ageing populations a global priority across research, policy and practice [1,2]. The number of people aged 60 years and older is projected to rise from 1.1 billion in 2023 to 1.4 billion by 2030, and by 2050, older adults will make up 22% of the world’s population [1]. This population growth is accompanied by rising rates of chronic illness and functional disability worldwide [3], with multimorbidity affecting 48% of those aged 59–73 years and 67% of those aged 74 years and above [4]. There is a growing awareness among nations of the need to monitor healthy ageing to reduce chronic conditions and functional decline among older adults [5,6]. Targeting healthy ageing through therapeutic or lifestyle interventions may help preserve function across the life course and support recovery from early functional decline [6].

The World Health Organization (WHO) defines healthy ageing as the process of developing and maintaining the functional ability that enables well-being in older age [7]. This definition deviated from the traditional deficit-based paradigm in ageing to focus on the functional ability of older adults. Functional ability refers to the capabilities that allow people to be and do what they value, even in the presence of chronic health conditions and disabilities [6]. It comprises an individual’s intrinsic capacity, the characteristics of their environment and the interaction between the two [8]. Intrinsic capacity refers to the aggregate of an individual’s physical and mental capacities, encompassing abilities such as locomotor, cognition, sensory, psychological and vitality. Intrinsic capacity is dynamic and influenced by genetic factors, physiological changes, lifestyle behaviours, social, economic, political, and environmental conditions across the life course [[9], [10], [11]].

There is a significant effort to improve the measurement of intrinsic capacity and functional ability as part of the United Nations Decade of Healthy Ageing [12,13]. To improve the consistency of how healthy ageing is measured worldwide, the WHO conducted a series of systematic reviews that sought to clarify core conceptual constructs and inform the selection of appropriate measures of intrinsic capacity across populations [12,14]. The systematic review that examined the measurement properties of instruments used to measure aspects of psychological capacity reported that psychological capacity still lacks a universally accepted operational definition [15].

Measures to capture depression (e.g., the Centre for Epidemiological Studies Depression Scale) and sleep have been predominantly used in validation studies of intrinsic capacity as proxy measures of psychological capacity [16,17]. However, the use of these tools has not been justified in terms of how it aligns with the intrinsic capacity framework, which prioritises capacity and strengths rather than deficits, such as poor sleep or reduced mental well-being. There is a need for clearer conceptualisation and validated scales for the domains of intrinsic capacity [18]. Psychometric evaluations of intrinsic capacity domains further reveal underreporting of content validity, construct validity and reliability in the psychological capacity domain, leaving it less standardised compared to other intrinsic capacity domains [19].

A study to develop and validate a measure of psychological capacity that aligns with the WHO framework of healthy ageing is currently underway. The first phase of the study was to develop an international consensus on the concept of psychological capacity. For this purpose, an international working group (IWG) was convened. A rapid scoping review was conducted ahead of the IWG to explore existing definitions and attributes of psychological capacity to inform the IWG discussion [20]. We report here the outcomes of the IWG.

## 2 Objectives

The overall purpose of the IWG was to develop a proposed working definition, conceptual framework, and identify attributes of psychological capacity, drawing on findings from the previous rapid review [20], to advance its measurement and monitoring. The objectives were to:

1 Review existing attributes, theoretical frameworks, and definitions related to psychological capacity.
2 Discuss and refine a conceptual framework relevant to psychological capacity.
3 Collaboratively develop a proposed working definition of psychological capacity and a list of potential attributes for the measure that can be applied across diverse cultural and research contexts.

## 3 Methods

This study adopted a qualitative, two-step discussion panel approach, guided by the WHO’s IWG [21]. The working group included two online meetings in which interactive discussions were held regarding psychological capacity in older age. Ethical approval for this study was obtained through Charles Sturt University (protocol number H25295).

### 3.1 Processes

Potential IWG participants were identified based on their expertise and publications in intrinsic capacity, gerontology, healthy ageing and measurement development [15,16,20,22]. In addition, experts were searched through the WHO Collaborating Centres Database by entering the subject term “ageing” [23]. An initial email invitation was sent to all identified experts (n = 66), representing the WHO six regions (Africa, America, Eastern Mediterranean, Europe, South-East Asia and Western Pacific). Those who did not respond to the initial invitation were sent two follow-up reminders, each approximately one week apart.

Prior to the first meeting, the experts who confirmed their participation were provided with a detailed information sheet, a draft of the rapid scoping review of psychological capacity attributes [20], the working group background paper outlining the IWG process, and a Consent Form. Participants provided consent via signed consent forms or email confirmation. Online meetings were held on 24 November and 4 December 2025, each lasting approximately 90 min. The meetings were chaired by the principal investigator (VI). Meetings were held, recorded and transcribed on Microsoft Teams, and supported by Padlet, an online interactive tool that allows participants to post ideas, comments and files in real time to facilitate shared input and discussion.

At the first meeting, the research team introduced the concepts of intrinsic and psychological capacity and outlined existing work in the field. Participants then discussed three guiding questions: (1) what psychological capacity means in the context of healthy ageing, (2) the key attributes and qualities that define it, and (3) the criteria for identifying these attributes. Attributes were conceptualised as measurable psychological characteristics or processes reflecting aspects of psychological capacity. Related challenges and future considerations were also explored.

Drawing on the discussion from the first meeting, the research team drafted a definition of psychological capacity and a conceptual framework. These materials were integrated into a summary report of the first meeting and circulated to participants before the second meeting.

During the second meeting, participants reviewed this summary report and further refined the proposed criteria and sub-domains of psychological capacity, with the discussion centred on agreeing on a draft conceptual framework and working definition and identifying any remaining challenges or discrepancies.

### 3.2 Analysis

All verbal discussions, Teams chat contributions, and Padlet inputs were documented and synthesised in NVivo-15. The transcripts were analysed using an iterative qualitative descriptive approach, focusing on identifying recurring concepts, areas of consensus and disagreement, and issues requiring further clarification. Transcripts from both meetings were reviewed in full and narratively synthesised in relation to the key discussion prompts. The analysis was used to refine the working definition, conceptual framework, and proposed attributes. During the meeting, findings were presented. Structured discussion and categorisation exercises were used to achieve consensus on key interpretations and recommendations. A research team consensus meeting was held to review the qualitative findings. Where statements or interpretations were unclear, these were checked against the original transcripts and meeting recordings, as well as chat or Padlet inputs, to ensure accurate representation of participants’ views.

## 4 Results

### 4.1 International working group participants

**Fig. 1:**
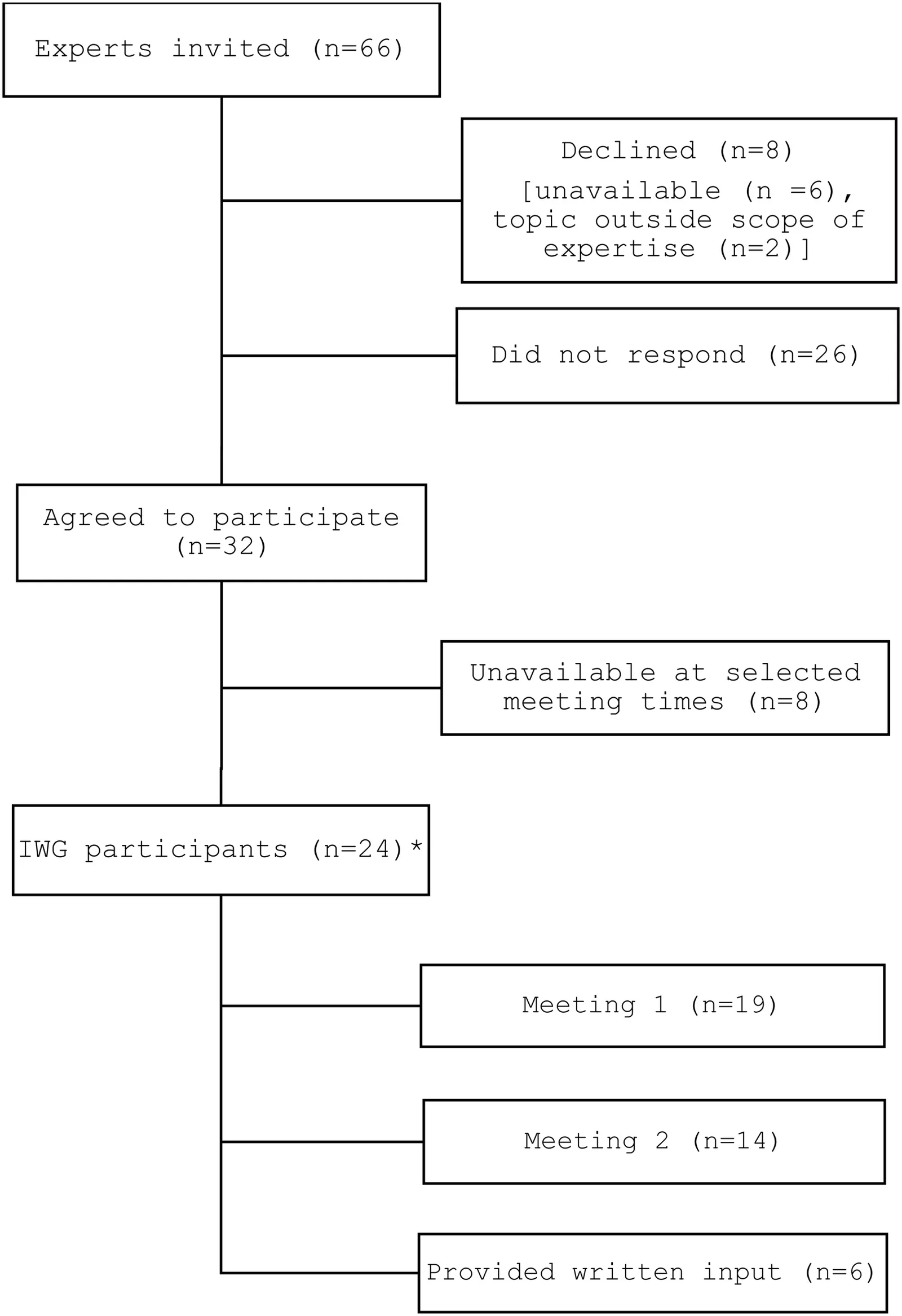
The International Working Group participant recruitment outcomes. * Numbers do not sum to 24, as some participants attended more than one meeting and/or also provided written input

Of the 66 experts identified, 24 participated in the meetings and/or provided written or verbal input out of session. Fig. 1 illustrates participant recruitment outcomes.

Participants’ fields of expertise included ageing, geriatrics, psychiatry, psychology, social work, and measurement science. Table 1 shows participants’ demographic characteristics.

**Table 1:** IWG participant characteristics.

Characteristics	Number	%
Gender		
Male	10	41.7%
Female	14	58.3%
WHO Region		
Africa	3	12.5%
America	6	25%
Eastern Mediterranean	1	4.2%
Europe	7	29.1%
South-East Asia	3	12.5%
Western Pacific	4	16.7%

The results below are presented in relation to the aims of (1) developing a working definition and conceptual framework, and (2) identifying the attributes of psychological capacity. Considerations and challenges for measuring psychological capacity identified by IWG participants are also discussed.

### 4.2 Developing a working definition and conceptual framework of psychological capacity

The primary purpose of the IWG process was to develop a draft definition of psychological capacity for healthy ageing. The first meeting involved a broad discussion around concepts of psychological capacity. Key aspects of the discussion are summarised below.

#### 4.2.1 Multi-dimensional construct

Preliminary discussion centred on clarifying the concept of intrinsic capacity as a combination of internal, individual-level attributes that support functioning and the ability to carry out valued activities. The broad and tangential discussion clearly illustrated that psychological capacity is a complex, multidimensional construct. It was felt to be broader and more complex than other intrinsic capacity domains, making boundary setting and differentiation from related constructs difficult.

#### 4.2.2 Distinguishing psychological capacity within the broader conceptual framework of intrinsic capacities and functional ability

Much of the discussion around defining psychological capacity was framed in terms of what it is not and distinguishing it from other key concepts such as well-being (an outcome), functional ability (which is determined by the interaction between intrinsic capacity and contextual factors) and cognitive capacity.

Participants noted the interconnection between the psychological and cognitive domains in the WHO intrinsic capacity framework. It was reported that judgment, decision-making and comprehension overlap with psychological processes, and late-onset mental disorders may signal early dementia, where neurodegenerative changes influence mood and behaviour. The discussion highlighted that cognitive decline could affect the expression of psychological attributes and that strong psychological resources cannot prevent symptoms caused by neurological disease. Participants debated whether psychological capacity should remain conceptually distinct from cognition or serve as an umbrella term for psychological, cognitive and social processes. Achieving clarity on this distinction was viewed as a major conceptual challenge, noting that it may be difficult to entirely separate psychological capacity from cognitive capacity, given the complex interplay between these capacities and abilities.

#### 4.2.3 Life course approach

Participants discussed whether the definition should explicitly focus on adaptation to age-related changes. There was broad agreement that it should have a broader framing and not be confined to later life contexts, noting that psychological capacity develops over the life course, shaped by personal histories, experiences and environmental conditions. There was discussion about the varying degrees of stability and changeability of psychological capacity attributes, and how these manifest as varying abilities in different life contexts. As such, it was noted that attributes included in the construct should be measurable and capable of change over time.

### 4.3 Psychological capacity proposed working definition and conceptual framework

Based on the first IWG meeting, the research team drafted a conceptual framework and preliminary working definition of psychological capacity (Appendices B–C). These were circulated to participants before the second meeting for review and refinement. Proposed conceptual framework

**Fig. 2:**
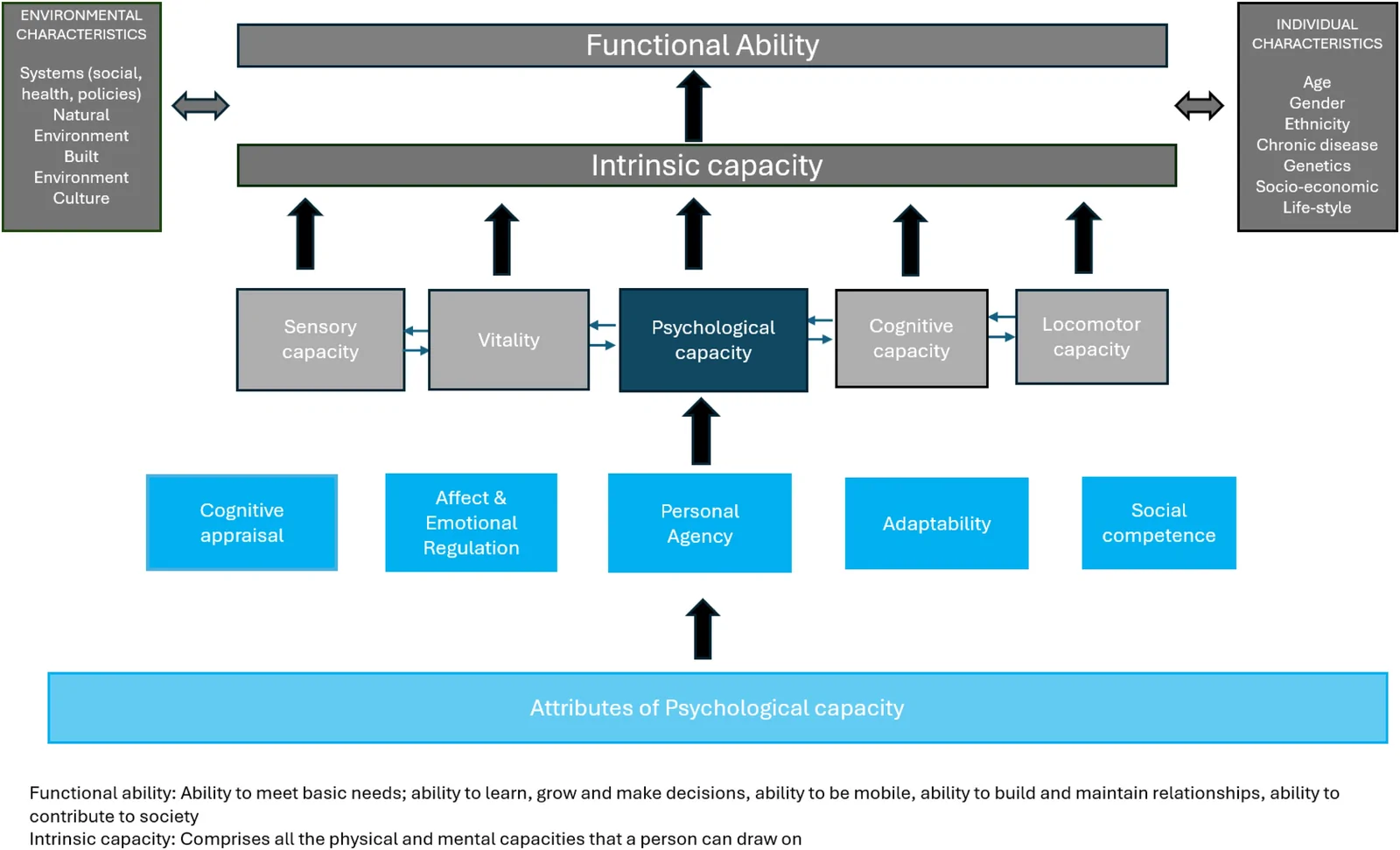
Revised conceptual framework for psychological capacity.

Participants commented on the proposed framework that illustrates the relationships between functional ability, intrinsic capacity and psychological capacity, and the key sub-domains of psychological capacity. The revised framework is presented in Fig. 2, with the earlier version provided in Appendix B. The group noted that the proposed framework aligns with the WHO intrinsic capacity model. However, they highlighted the need for stronger alignment between the working definition of psychological capacity and its representation in the framework. Therefore, terminology was refined to ensure consistency.

#### 4.3.1 Working definition of psychological capacity

The revised working definition of psychological capacity is presented in Box 1, with the preliminary version provided in Appendix C. During the second IWG meeting, participants discussed several aspects of the preliminary definition that required clarification or refinement.

There was broad agreement that psychological capacity should be defined as an intrinsic, individual-level construct, distinct from functional ability and separate from social and environmental factors; however, there were concerns that the definition did not adequately acknowledge social components, and the alternative term “psychosocial capacity” was suggested. This terminology is not congruent with the WHO intrinsic capacity framework, so it was considered unsuitable for broader adoption; however, the significance of social functioning and related underlying intrinsic capacities to healthy ageing was noted.

The term “state-like”, originally used to distinguish dynamic or changeable characteristics from trait-like attributes, was queried and considered unnecessary and potentially misleading, given the importance of changeability and responsiveness to intervention. At the same time, participants noted that relatively stable characteristics, such as personality traits, may influence psychological capacity and should not be excluded by definition.

The term “personal agency” raised concerns about conceptual overlap with learning and decision-making, as well as cultural limitations. Some participants argued that the term may reflect Western individualistic views and proposed alternatives such as life purpose, attachment to life or reflective integration. The term “psychological potential” was also viewed as difficult to operationalise, with suggestions to focus on measurable psychological attributes.

Box 1. The revised working definition of psychological capacity

“Psychological capacity describes all the psychological attributes that contribute to an individual’s functional ability. It comprises intrinsic psychological characteristics, assets and skills that enable the individual to respond to internal stressors and contextual demands. These capacities are expressed as flexible attributes that enable cognitive appraisal, affect and emotional regulation, adaptability, coping, social competence, and personal agency.”

#### 4.3.2 Sub-domains of psychological capacity

Findings from the rapid review and discussions during the first IWG meeting, the research team identified and conceptually defined five sub-domains of psychological capacity and working definitions thereof. Note that several attributes can be considered to have conceptual relevance to more than one sub-domain.

*Cognitive appraisal* refers to how individuals interpret and assign meaning in a situation. *Personal agency* is the ability, beliefs and expectations that enable individuals to make choices and take actions that influence their lives. *Affect and emotional regulation* involve recognising, understanding and regulating emotions. *Adaptability* refers to adjusting thoughts, emotions and behaviours in response to challenges or adversity. *Social competence* consists of skills that enable effective, appropriate and comfortable interaction with others.

It was proposed that adaptability and personal agency could be merged into a single sub-domain, given their shared role in supporting selection, optimisation and compensation strategies in later life. The IWG further noted challenges in defining the emotional sub-domain, particularly given the historical use of depression as a proxy measure. It was noted that previous reliance on depression had been a consequence of data constraints rather than conceptual fit. The broader challenge of separating intrinsic psychological attributes from outcome measures was also noted. Participants emphasised that individuals living with depression may still demonstrate effective emotional processing and functioning, depending on context and other capacities.

Distinguishing emotions and affect from the presence or absence of a mental disorder was seen as essential. However, participants discussed how focusing only on positive affect risks ceiling effects and does not capture the processes that support functioning across different circumstances.

### 4.4 Refining the attributes of psychological capacity

Alongside developing the definition of psychological capacity, another key aim for the IWG process was to determine a draft list of attributes of psychological capacity (for later confirmation in a modified Delphi process). In the first meeting, participants engaged in an open discussion about a wide range of attributes and related considerations. In the second meeting, the discussion of attributes was assisted by the development of criteria for inclusion and sub-domains, which are discussed further below.

#### 4.4.1 Attributes’ inclusion criteria

Based on the discussion at the first IWG meeting, the research team drafted criteria for psychological capacity attributes as follows: (1) individual-level attribute, (2) stable but flexible, (3) not an outcome, (4) unique (not present in other intrinsic capacity domains), (5) can build and maintain functional ability, and (6) defined at the level of underlying psychological processes (not biological mechanisms or symptom manifestations).

#### 4.4.2 Attributes discussion

Across the two IWG meetings, there was rich discussion about some of the attributes identified in the rapid review. Attributes related to adaptation, problem solving and emotional stability were central, including coping abilities, resilience, flexibility, acceptance, hope, optimism, self-efficacy, mastery and motivation. Resilience was described as multidimensional and viewed as central to psychological capacity.

Participants also suggested and discussed other attributes that had not been identified in the rapid review. Locus of control was discussed in depth. While an internal locus of control was seen as beneficial, participants noted that control can also operate through external supports. Participants also noted that some attributes, such as locus of control and self-efficacy, may vary across situational contexts such as work, family and leisure.

Connectedness was also a recurring theme. Participants distinguished between intrapersonal connectedness (self-understanding and acceptance) and interpersonal connectedness (relationships and belonging). They noted that the number of social connections is external to the individual and therefore ‘social connections’ does not qualify as an attribute of psychological capacity, according to the criteria described above. Instead, it was suggested focusing on constructs linked to personal agency, such as social capacity or satisfaction with relationships. Social competency, including the ability to interact effectively and find one’s place in the community, was also emphasised. Participants highlighted the need to recognise older people’s social preferences, including the wish to limit social engagement.

Further attributes discussed by IWG participants included positive affect, emotional regulation, emotional intelligence and affect processing. Reflective and deliberative abilities such as judgement, decision making and the capacity to form and revise goals were also considered important. Spirituality, religion and purpose in life were identified as meaningful attributes in many cultural contexts. Ageing-related elements, such as internalised ageism and purpose after retirement, were also proposed. Wisdom was discussed as spanning cognitive appraisal and social cognition, although it may function more as a stable trait than a capacity.

Sleep was suggested as a possible attribute because it had previously been used as a proxy indicator for psychological capacity (as discussed earlier). There was debate on whether sleep aligns more with vitality, psychological capacity or a separate construct, and whether, as a biological need, it should be viewed as a behaviour, an outcome or a foundational contributing factor rather than an intrinsic capacity.

The IWG participants also identified a set of attributes for possible exclusion due to overlap with cognitive capacity or performance level functioning. These included planning and structuring tasks, deliberation, decision-making and judgment, perception and comprehension, rational thinking, memory and recall and internalised ageism.

Based on this discussion, an updated list of psychological capacity attributes was generated, categorised by sub-domain (personal agency, cognitive appraisal, affect, adaptability, social competence) and presented in Appendix D.

### 4.5 Considerations and challenges for measuring psychological capacity

In addition to the conceptual considerations relating to psychological capacity definition and attributes, several broader measurement considerations were discussed.

#### 4.5.1 Clear purpose and broad utility

Participants emphasised the need for a clear definition that can support different measurement approaches across settings. Participants further stressed the need to clarify the intended purpose of the measure. Tools for population-level monitoring differ from those intended for individual care planning, and these purposes require different types of information.

The group emphasised that any measure of psychological capacity should be brief and practical so that it can be used for self-report or administered by non-specialists in both research and clinical settings.

Participants noted that existing research has largely relied on secondary datasets and proxy indicators not specifically designed to assess psychological capacity, with inconsistent operationalisation across studies limiting comparability. They emphasised the need for a measure that is comparable and meaningful across age groups and contexts. Cultural and social variation was emphasised as an important consideration. Attributes may differ across cultures, social strata and community settings, and the workplace and transition to retirement were noted as particularly influential.

#### 4.5.2 Target population and relevance across the life-course

The group furthermore discussed whether psychological capacity can be consistently measured across different ages. It was noted that certain indicators may be age-dependent and therefore recommended using broader indicators to support comparability across contexts. Participants acknowledged that psychological capacity develops across the life course and should not be conceptually restricted to a particular age group. However, given the focus on psychological capacity within the intrinsic capacity and healthy ageing framework, it was suggested that the initial measure should focus on middle-aged and older adults. Regardless, participants cautioned against a ‘successful ageing’ framing and instead recommended adopting benchmarks that are realistic and achievable.

#### 4.5.3 How to measure capacity rather than functional outcomes

Participants raised concerns about the strong reliance of existing psychological instruments on functional outcomes. Many measures are validated against functioning and include functional items, which limit their suitability for assessing intrinsic psychological capacity.

More specifically, the group highlighted the difficulty of separating psychological capacity, defined as internal attributes, from functional ability, which reflects how individuals interact with their environment. Using instruments that incorporate functional impairment (such as depression scales) risks conflating the two constructs and creates circularity when psychological capacity is examined as a predictor of functioning.

In addition, participants questioned whether psychological capacity can be meaningfully measured at the level of underlying processes such as appraisal, regulation and adaptation without relying on outcome-based items, noting this as a central challenge for instrument development.

## 5 Discussion

An IWG comprising 24 experts across six WHO regions participated in two online meetings and developed the proposed working definition of psychological capacity. Psychological capacity was identified as an intrinsic, multidimensional construct supporting adaptation, emotional regulation, cognitive appraisal, social competence and personal agency across the life course. It is conceptualised as an underlying set of psychological attributes of an individual that promotes well-being and functional ability and is distinct from mental health symptoms or behaviours. A refined definition and criteria for attribute inclusion were established to ensure alignment with the intrinsic capacity model and to focus on underlying psychological processes rather than symptoms or behavioural manifestations.

### 5.1 Psychological capacity - Definition

Psychological capacity was considered distinct from other intrinsic capacity domains in terms of how the construct could be defined and measured. For example, psychological capacity may not show age-related decline, unlike other intrinsic capacities [24,25] and may not be measured using performance-based measures [26,27].

There were a few key arguments around how the construct could be defined. First, psychological capacity should adequately reflect underlying intrinsic psychological processes, rather than their expression in everyday life. Intrinsic capacity refers to an individual's physical and mental capabilities, whereas functional ability reflects how these capacities are realised through interactions with personal and environmental factors [6,7]. Measures such as loneliness, well-being or depressive symptoms therefore represent outcomes or manifestations of functioning rather than intrinsic psychological capacity. Although reduced psychological capacity would increase vulnerability to depression or loneliness, these experiences arise through the complex interaction of biological, psychological, social and environmental influences. To date, psychological capacity has often been operationalised using depression scales or sleep indicators [28,29]. While these proxy measures have enabled empirical research on intrinsic capacity, they may not fully capture the underlying construct of psychological capacity [30].

Second, psychological capacity should be stable yet modifiable through intervention. For instance, Psychological Capital, a stake-like higher-order construct comprising hope, efficacy, resilience and optimism, has been demonstrated to be amenable to targeted interventions aimed at improving performance and well-being [31]. However, the evidence is limited to work settings, and the concept has not been adequately explored in ageing [20].

Third, psychological capacity should be conceptualised along a continuum of regulatory and adaptive processes rather than as an idealised state, consistent with life-course and process-oriented models of adaptation. Lifespan developmental theory indicates that later-life functioning reflects cumulative processes shaped by earlier adaptation and exposure [32,33]. Process-oriented models conceptualise functioning as the result of ongoing self-regulatory processes, such as goal selection, optimisation and compensation, through which individuals actively adjust to changing opportunities and limitations over time. Within this framing, later life psychological functioning reflects cumulative patterns of adaptation shaped by earlier exposures and regulatory strategies rather than a fixed or optimal end state [34].

Finally, psychological capacity should contribute uniquely to intrinsic capacity despite conceptual and empirical overlap with other capacities. For example, memory and executive functions follow distinct developmental trajectories [35] to emotional and motivational processes, which may remain stable or improve [36]. Therefore, the proposed definition and framework distinguish psychological capacity from cognition for conceptual and measurement purposes, while acknowledging the interaction between the two domains.

### 5.2 Conceptual framework and attributes

Psychological capacity is part of intrinsic capacity and is situated within a broader context of individual characteristics, environmental influences and their interaction with intrinsic capacity to determine functional ability [37]. The proposed conceptual framework reflects psychological capacity as a multidimensional construct determined by cognitive appraisal, affect and emotional regulation, adaptability, personal agency and social competence. The interaction between these sub-domains builds the overall psychological capacity of an individual that contributes to functional ability. These sub-domains were developed by categorising the attributes derived from a rapid review of psychological attributes and IWG [20].

The cognitive appraisal sub-domain refers to a person’s capacity to evaluate and interpret a situation in relation to goals, values and perceived coping resources. Positive cognitive attributes such as hope, optimism and purpose can be understood as cognitive motivational orientations that shape appraisal processes. By supporting expectations of agency, achievable goals and favourable future outcomes, these attributes promote challenge-focused rather than threat-focused interpretations of life situations [38] and are uniquely associated with reduced incidence of disease and improved well-being [39,40].

The affect and emotional regulation sub-domain refers to the capacity of the person to recognise, understand and regulate emotions [41]. Emotional regulation may be maintained or improved in later life and reflects the interaction of multiple biological, cognitive and interpersonal processes [42,43]. Decision-making may increasingly reflect emotion regulation processes even if cognitive resources diminish [44].

The personal agency sub-domain refers to the capacity of the individual to make choices and take actions to influence their life, including the ability to choose and feel in control of actions and pursue meaningful goals [45]. Complementing this sub-domain, the adaptability domain refers to the competence of the individual to handle significant changes by rebounding from adversity or uncertainty [46]. Within this sub-domain, resilience as a psychological attribute of development and maintenance of healthy adaptation has been robustly related to healthy ageing [47].

Finally, the social competence sub-domain was added after deliberation by the IWG. Social capacities can be situated within psychological capacity when conceptualised as individual-level assets such as social cognition, social intelligence and empathy. These processes support interpersonal functioning and are embedded in affective, motivational and self-regulatory systems that shape appraisal and behavioural responses [48].

Various attributes may contribute to each of these domains. We have identified 42 attributes of psychological capacity to progress to a Delphi study for further assessment [49]. There was a general agreement on the relevance of the attributes to psychological capacity for further consideration in the Delphi study, while a few were debated. For example, sleep was debated as a potential indicator of psychological capacity but also recognised as reflecting broader biological reserve. Another example was personality function. Personality function may contribute as another potential sub-domain of psychological capacity; however, a clear consensus was not met in the IWG. Personality traits are strong predictors of ageing outcomes but are typically conceptualised as relatively stable dispositions [50,51], whereas a capacity-based framework implies dynamic and potentially modifiable processes [52]. Aspects of personality functioning, such as self-regulation and interpersonal effectiveness, overlap conceptually with psychological capacity, making the distinction less straightforward. Given the lack of consensus around these issues, sleep and personality will be included in the Delphi study to further explore their relevance to psychological capacity.

### 5.3 Challenges to measurement

Several challenges need to be considered in the development of a measure of psychological capacity. The three critical points were

a) Purpose of the measure: The value of a psychological capacity measure depends on clarity about its intended purpose. Instruments for population monitoring require cross-cultural comparability to enable meaningful comparisons across populations and settings, as well as longitudinal sensitivity to capture changes in psychological capacity over time [53]. Clinical tools, however, may need empirical validation to support risk identification and care planning [13,54]. A brief and practical measure may also have utility in community and public health settings, for example, to inform healthy ageing programmes and population-level planning. These different applications may place different demands on the measure. Without this distinction, measures risk being too complex for surveillance or too limited for research and clinical use [55,56].
b) Cultural adaptations: Cultural and regional variations are central to defining psychological capacity as well. While the underlying construct may be universal, its expression and relative importance may differ across social and economic contexts. Constructs such as agency, purpose, connectedness and emotional regulation are shaped by cultural norms around autonomy and interdependence [57]. This has implications for measurement, as items developed in individualistic settings may not function equivalently in collectivist or resource-constrained contexts [58]. As noted in the intrinsic capacity measurement literature, cross-cultural validity requires careful attention to language, interpretation and contextual relevance, particularly for global monitoring [15,59].
c) Ceiling effect: While some participants indicated mental health symptoms should not be used as indicators of psychological capacity, they also cautioned against an exclusively positive framing. This reflects broader debates in health and psychology research, where overly positive indicators have been criticised for producing ceiling effects in healthier populations and limiting sensitivity to clinical change [60,61].

### 5.4 Strength and limitations

The IWG discussion reported here represents an initial step in the development of a conceptual framework for psychological capacity. Its strength was the inclusion of experts from all six WHO regions, with multidisciplinary perspectives spanning ageing, psychology and public health, which enabled a diverse debate. Although participants represented different WHO regions, not all ethnic groups were represented; therefore, perspectives across all ethnic and cultural groups may not have been fully captured. In addition, participation varied across regions and time zones, and levels of engagement differed, with some contributors providing written feedback rather than attending all discussions. Particularly, the participation of consumers, i.e., older adults or their proxy, has not been fully considered in the early stage of discussion. The framework, therefore, reflects an early stage of consensus and requires further refinement through formal Delphi procedures and broader international representation. Conceptual challenges remain, particularly in distinguishing psychological capacity from functions and outcomes, underscoring the need for clearer operational definitions and empirical testing of candidate sub-domains.

## 6 Conclusions

Based on discussions within the IWG, the research team developed a working definition and conceptual framework for psychological capacity. It was defined as an intrinsic, multidimensional construct comprising internal psychological assets and skills that support appraisal, emotional regulation, adaptability, social competence and personal agency across the life course. Psychological capacity was distinguished from functional ability, well-being and mental health symptoms, while acknowledging conceptual overlap with cognitive and social processes. The framework establishes criteria for attribute inclusion and identifies measurement challenges, including domain boundaries, ceiling effects, lifespan variation and cross-cultural applicability. These outputs provide a structured foundation for psychological capacity as a core component of healthy ageing. Establishing a shared conceptual understanding is an essential prerequisite for the development and validation of a standardised measure of psychological capacity.

## Contributions

VI, CO, SH, and JK conceived the study, obtained funding and oversaw its design and implementation. NH, JAT and VI conducted the data collection and facilitated the working group meetings, which were chaired by VI. IWG members contributed to the discussion. AW and NH collated, synthesised and analysed the data, and prepared the interim reports. NH led the drafting of the manuscript. NH, AW, VI, CO, SH, JK reviewed and edited the manuscript. All authors have read and approved the final version for submission.

## Declaration of Generative AI and AI-assisted technologies in the writing process

We have not used Generative AI or AI-assisted technologies in the writing process.

## Role of funding source

The funding source had no involvement in this study.

## Data statement

The data comprise transcripts of the IWG meetings. Due to the potentially identifiable nature of these qualitative data and the conditions of participant consent and ethical approval, the transcripts are not publicly available.

## Declaration of competing interest

We have no known conflict of interest to disclose.
