# Supplementary material for: Definition and conceptual framework of the psychological domain of intrinsic capacity in healthy ageing: outcomes from the MOSAIC International Working Group
Source: J Nutr Health Aging. 2026 Aug 28;30(11):100969. doi: 10.1016/j.jnha.2026.100969 (PMC13545368; doi:10.1016/j.jnha.2026.100969)
Supplement: Supplementary file 1 [file mmc1.docx]

**Definition and conceptual framework of the psychological domain of intrinsic capacity in healthy ageing: outcomes from the MOSAIC International Working Group: Appendices**

Table of Contents

[Appendix A. Members of the International Working Group by Alphabetical order	2](#_Toc229559872)

[Appendix B. Draft conceptual framework	3](#_Toc229559873)

[Appendix C: Full list of attributes of psychological Capacity and definitions considered in the International Working Group	4](#_Toc229559874)

# Appendix A. Members of the International Working Group by Alphabetical order

Aarti Jagannathan

Andrea B. Maier

Athula Sumathipala

David Beckwée

Eduardo Ferriolli

Fiona Alpass

Huali Wang

Jan Boehnke

John R. Beard

Leon Geffen

Liat Ayalon

Luis Miguel Gutierrez-Robledo

Margie Lachman

Marilyne Menassa

Matthew Prina

Muthoni Gichu

Philipe de Souto Barreto

Rebecca Robbins

Robert Stewart

Saman Khalatbari-Soltani

Sebastiana Kalula

# Appendix B. Draft conceptual framework

**Figure S1** illustrates the conceptual relationship between functional ability, intrinsic capacity, and psychological capacity, as well as the key attributes of psychological capacity. It was developed based on discussions during the first meeting and informed by findings from the rapid review. The structure reflects the WHO framework for intrinsic capacity, with psychological capacity positioned as a core component alongside sensory, cognitive, locomotor, and vitality capacities. The selected attributes are part of psychological processes such as cognitive appraisal, emotional adjustment, adaptability, social mechanisms and personal agency.

**Figure S1. Proposed conceptual framework for psychological capacity**

# Appendix C: Full list of attributes of psychological Capacity and definitions considered in the International Working Group

**Table 1. List Attributes and definitions categorised by sub-domain (personal agency, cognitive appraisal, affect, adaptability, social competence).**

| Sub-domain | Attribute | Formal definition | Short definition | Source | Include | Reason for exclusion |
| --- | --- | --- | --- | --- | --- | --- |
| Adaptability | Adaptability | Adaptability refers to an individual’s capacity to adjust their cognitive, behavioural, and emotional responses to effectively manage new, changing, or uncertain situations. | Being able to change how you think, act, and feel when situations shift. | IWG discussion | yes |  |
| Adaptability | Coping | Coping is defined as constantly changing cognitive and behavioural efforts to manage specific external and/or internal demands that are appraised as taxing or exceeding the resources of the person. | Thoughts and actions are used to manage stressful or demanding situations. | IWG discussion | yes |  |
| Adaptability | Flexibility | The ability to adapt behaviour and thinking in response to changing environmental contingencies. | Being able to adapt thoughts and actions to changing situations. | Rapid review | yes |  |
| Adaptability, Cognitive appraisal, Personal agency, Affect | Resilience | Our ability to bounce back from life’s challenges and unforeseen difficulties, providing mental protection from emotional and mental disorders. | The ability to bounce back from setbacks and cope with difficulties. | Rapid review | yes |  |
| Affect | Affect processing/ regulation | affect regulation)- the attempt to alter or control one’s mood or emotional state to maximise pleasant experiences and minimise unpleasant ones. Because people cannot usually change their emotions simply by deciding to feel differently, they use many indirect strategies for affect regulation. These include cognitive techniques such as reframing and distraction, behavioural methods such as progressive relaxation and meditation, and unconscious processes such as denial and dissociation. | Managing mood to enhance pleasant emotions and lessen unpleasant ones. | Rapid review | yes |  |
| Affect | Emotional intelligence | Emotional intelligence involves the ability to perceive accurately, appraise, and express emotion; the ability to access and generate feelings when they facilitate thought; the ability to understand emotion and emotional knowledge; and the ability to regulate emotions to promote emotional and intellectual growth. | Recognising and understanding emotions, and using them to guide thinking and behaviour | Rapid review | yes |  |
| Affect | Emotional regulation | Emotion regulation refers to the processes by which individuals influence which emotions they have, when they have them, and how they experience and express these emotions. | Managing when emotions arise and how they are felt or expressed | Rapid review | yes |  |
| Affect | Self-regulation | Self-regulation refers to the capacity to alter one’s responses, including thoughts, emotions, and behaviours, in order to pursue goals and conform to standards. | The capacity to adjust thoughts, emotions, and behaviours to pursue goals or meet expectations | Rapid review | yes |  |
| Affect | Insight | Insight is the clarity of understanding of one’s thoughts, feelings, and behaviours. (Insight ) An awareness of underlying sources of emotional, cognitive, or behavioural responses and difficulties in oneself or another person (APA). | Understanding the sources of your thoughts, feelings, behaviours, and vulnerabilities | Rapid review | yes |  |
| Affect | Mindfulness | Paying attention in a particular way: on purpose, in the present moment, and nonjudgmentally; Awareness of one’s internal states and surroundings. The concept has been applied to various therapeutic interventions- for example, mindfulness-based cognitive behaviour therapy, mindfulness-based stress reduction, and mindfulness meditation- to help people avoid destructive or automatic habits and responses by learning to observe their thoughts, emotions, and other present-moment experiences without judging or reacting to them (APA). | Being aware of your thoughts, feelings, and surroundings in the present moment without judging them. | Rapid review | yes |  |
| Affect, Cognitive appraisal | Acceptance | Acceptance involves the active and aware embrace of private events without unnecessary attempts to change their frequency or form, especially when doing so would cause psychological harm. | Allowing thoughts, feelings, situations, and behaviours to be present without struggling against them in unhelpful ways. | Rapid review | yes |  |
| Personal agency | Autonomy | Autonomy refers to the experience of volition and psychological freedom in one’s actions, such that behaviour is self-endorsed rather than controlled. | Experiencing choice and freedom in your actions, so behaviour feels self‑directed. | Rapid review | yes |  |
| Personal agency | Motivation | Energy, direction, persistence, and equifinality- all aspects of activation and intention. Refers to the process that initiates, guides, and maintains goal-directed behaviour. | Wanting to do something and putting in effort to achieve it. | Rapid review | yes |  |
| Personal agency | Perseverance | The continued effort toward long-term goals despite obstacles, setbacks, or discouragement. | Keeping up the effort toward long-term. goals even when facing setbacks | Rapid review | yes |  |
| Personal agency | Persistence | The continued repetition of a behaviour or effort in the face of obstacles or lack of immediate success. | Keeping up effort despite obstacles or no immediate results. | Rapid review | yes |  |
| Personal agency | Proactivity | Anticipatory, change-oriented, and self-initiated behaviour in the workplace; The tendency to take initiative, anticipate future challenges, and act in advance to influence outcomes. | Taking initiative and acting ahead of future challenges. | Rapid review | yes |  |
| Personal agency | Responsibility/ attribution of responsibility | The perceived causality and accountability for an outcome. | Seeing an outcome as caused by, and accountable to, a person or action. | Rapid review | yes |  |
| Personal agency | Self-responsibility | an individual’s accountability for their own actions and future plans, based on personal autonomy and self‑determination. It includes taking ownership of one’s wellbeing, decisions and behaviour through self-awareness and self-regulation. | Actively taking ownership of behaviour and its consequences. | Rapid review | yes |  |
| Personal agency | Self-efficacy | The beliefs in one’s capabilities to organise and execute the courses of action required to produce given attainments. | Belief in one’s ability to plan and carry out actions to achieve desired outcomes. | Rapid review | yes |  |
| Personal agency | Self-Mastery | The extent to which one regards one’s life chances as being under one’s own control, in contrast to being fatalistically ruled. | Seeing your life outcomes as under your own control rather than determined by fate. | IWG discussion | yes |  |
| Personal agency | Sense of control | Perceived control- the belief that one can determine one's own internal states and behaviour, influence one's environment, and/or bring about desired outcomes. | Believing you can influence your life, environment, and outcomes. | IWG discussion | yes |  |
| Personal agency, Affect, Social competence | Personality functioning | an individual's core capacity for self-regulation, identity, and interpersonal relationships, representing a continuum from adaptive to dysfunctional. It acts as a primary diagnostic criterion in DSM-5 and ICD-11 to determine the severity of personality disorders by measuring how a person navigates their internal and external world. | How a person understands themselves, manages emotions and goals, and relates to others. | Rapid review | yes |  |
| Cognitive appraisal | Capacity to register (encoding) | Encoding)- the processes by which information is registered and transformed into a form suitable for storage in memory. | The process of taking in information and converting it into a form that can be stored in memory. | IWG discussion | yes |  |
| Cognitive appraisal | General self/world comprehension | The process by which individuals construct a coherent mental representation of information by integrating input with prior knowledge; Comprehension- the act or capability of understanding something, especially the meaning of a communication (APA). | The ability to notice, interpret, and make sense of information from the world. | Rapid review | yes |  |
| Cognitive appraisal | Internalised ageism | The process by which older individuals adopt and apply negative age-related stereotypes and attitudes to themselves. | Older people internalising and applying negative age-related stereotypes to themselves. | IWG discussion | yes |  |
| Cognitive appraisal | Rational thinking | The use of analytic reasoning processes that operate according to rules of logic, probability, and evidence evaluation; Thinking according to logical rules, which is considered by many theorists to be a central part of intelligence (APA). | Using logical, evidence‑based reasoning to make decisions and judge information. | Rapid review | yes |  |
| Cognitive appraisal | Hope | Desire accompanied by the expectation of obtaining what is desired or the belief that it is obtainable (dictionary definition). | Wanting something and believing it can happen. | Rapid review | yes |  |
| Cognitive appraisal | Meaning in life | The sense made of, and significance felt regarding, the nature of one’s being and existence. | Feeling that life makes sense and has personal significance. | IWG discussion | yes |  |
| Cognitive appraisal | Optimism | The extent to which people hold generalised favourable expectancies for their future. | General expectation of positive outcomes. | Rapid review | yes |  |
| Cognitive appraisal | Purpose in life | Having goals, intentions, and a sense of direction that give life meaning. | Having goals and a sense of direction that give life meaning. | IWG discussion | yes |  |
| Cognitive appraisal | Self-esteem | a positive or negative attitude toward the self as a totality; The degree to which the qualities and characteristics contained in one’s self-concept are perceived to be positive. It reflects a person’s physical self-image, view of their accomplishments and capabilities, and values and perceived success in living up to them, as well as the ways in which others view and respond to that person. The more positive the cumulative perception of these qualities and characteristics, the higher one’s self-esteem. | How positively a person views their own qualities, abilities, achievements, and worth. | Rapid review | yes |  |
| Cognitive appraisal | Spirituality | A way of being and experiencing that comes about through awareness of a transcendent dimension and that is characterised by certain identifiable values regarding self, others, nature, life, and whatever one conceives as the Ultimate; A concern for or sensitivity to things of the spirit or soul, especially as opposed to materialistic concerns. More specifically, a concern for God and a sensitivity to religious experience, which may include the practice of a particular religion but may also exist without such practice. The fact or state of being incorporeal (APA). | Awareness of a deeper sense of connection or meaning beyond the material. | IWG discussion | yes |  |
| Cognitive appraisal, Personal agency | Self-coherence/ Sense of coherence | 1. A perception of having clarity or intelligibility, that is, of being capable of thinking and expressing oneself in a clear and consistent manner; 2. the ability to present a narrative of oneself in a way that is understandable and easy to follow. Sense of coherence is considered a protective factor against the deleterious effects of stress on physical and mental health (APA) | Being able to make sense of experiences and present the self in a clear, coherent way. | Rapid review | yes |  |
| Social competence | Assertion | A speech act by which a speaker puts a proposition forward as true, thereby adding it to the common ground of a conversation; The forceful statement of or insistence on one’s beliefs, claims, rights, or the like (APA). | Making a firm statement of one’s beliefs or claims. | Rapid review | yes |  |
| Social competence | Assertiveness | A form of communication in which individuals express their needs, rights, and opinions confidently while maintaining respect for others | Communicating needs and opinions confidently and respectfully. | Rapid review | yes |  |
| Social competence | Group integration | An individual’s perception of the closeness, bonding, and unity of the group as a collective. | Perception of closeness, bonding, and unity within the group. | Rapid review | yes |  |
| Social competence | Social capacity | The ability to understand social information, engage with others, and adapt one’s behaviour to social contexts in ways that support participation and relationships. | Understanding social cues, engaging with others, and adapting behaviour to social situations. | IWG discussion | yes |  |
| Social competence | Social cognition | How people think about themselves and others, including how they select, interpret, remember, and use social information. | How people understand and process information about themselves and others. | Rapid review | yes |  |
| Social competence | Social competence | The cognitive, emotional, and behavioural skills required to navigate social situations effectively and appropriately across contexts. | The cognitive, emotional, and behavioural skills that allow a person to handle social situations effectively and appropriately. | IWG discussion | yes |  |
| Social competence | Social skills | a set of learned abilities that enable an individual to interact competently and appropriately in a given social context. The most commonly identified social skills in Western cultures include assertiveness, coping, communication and friendship-making skills, interpersonal problem solving, and the ability to regulate one’s cognitions, feelings, and behaviour (APA). | Abilities that support effective and appropriate social interaction. | Rapid review | yes |  |
| Social competence | Empathy | The process by which one comes to understand the mental states of others by experiencing an inner imitation of their feelings; understanding a person from their frame of reference rather than one’s own, or vicariously experiencing that person’s feelings, perceptions, and thoughts (APA). | Understanding others by taking their perspective and sensing their feelings. | Rapid review | yes |  |
| Social competence | Trust | An expectancy held by an individual or a group that the word, promise, verbal or written statement of another individual or group can be relied upon; Reliance on or confidence in the dependability of someone or something. In interpersonal relationships, trust refers to the confidence that a person or group of people has in the reliability of another person or group; specifically, it is the degree to which each party feels that they can depend on the other party to do what they say they will do. The key factor is not the intrinsic honesty of the other people but their predictability. Trust is considered by most psychologists to be a primary component in mature relationships with others, whether intimate, social, or therapeutic (APA). | Expecting that another person or group will act reliably and keep their commitments. | Rapid review | yes |  |
| Adaptability | Endurance (psychological) |  |  | Rapid review | no | Included in resilience |
| Adaptability | Immediate adaptation |  |  | Rapid review | no | Included in resilience |
| Adaptability | Psychological potential |  |  | Rapid review | no | An umbrella term used in the definition of psychological capacity, not a measurable individual attribute? |
| Adaptability, Cognitive appraisal, Personal agency, Affect | Ability to overcome adversity/challenges |  |  | Rapid review | no | Not appear as a named construct in major psychological theories |
| Adaptability, Cognitive appraisal, Personal agency, Affect | Anti-frustration ability | (Distress tolerance): the perceived or actual ability to withstand negative emotional states, including frustration, without escaping or avoiding the stressor. |  | Rapid review | no | Included in resilience |
| Adaptability, Cognitive appraisal, Personal agency, Affect | Anti-setback ability |  |  | Rapid review | no | Not appear as a named construct in major psychological theories |
| Affect | Positive affect | The extent to which a person feels enthusiastic, active, and alert. |  | IWG discussion | no | No intrinsic, Temporary, outcome |
| Affect | Psychological aspects of emotional wellness | Emotional well-being refers to the ability to manage thoughts and feelings, cope with stress, and maintain satisfying relationships. |  | IWG discussion | no | Outcome |
| Affect | Anxiety | Anxiety is the anticipation of future threat, often accompanied by muscle tension, vigilance, and avoidance behaviour. |  | IWG discussion | no | Possibly not an intrinsic capacity, but an outcome? |
| Affect | Forgiveness |  |  | Rapid review | no | Behaviour |
| Affect | Patience |  |  | Rapid review | no | Behaviour |
| Personal agency, Cognitive appraisal | Capacity for Decision making | Decision-making capacity refers to a patient’s ability to understand relevant information, appreciate the situation and its consequences, reason about treatment options, and communicate a choice. |  | IWG discussion | no | Overlaps with cognitive capacity |
| Cognitive appraisal | Recall | 1. To transfer prior learning or past experience to current consciousness: that is, to retrieve and reproduce information; to remember.  2. The process by which this occurs (APA) |  | IWG discussion | no | Overlaps with cognitive capacity |
| Cognitive appraisal | Attachment to life | Overall attitude, perspective, or way of interpreting and responding to life events and circumstances. |  | Rapid review | no | Outcome; part of ‘hope’ |
| Cognitive appraisal | Flow experience |  |  | Rapid review | no | Outcome |
| Cognitive appraisal | Outlook on life |  |  | Rapid review | no | More of a descriptive phrase than a formally defined psychological construct |
| Cognitive appraisal | Realistic goals | Goals that are achievable given an individual’s current resources, abilities, and circumstances, and that align with their values and priorities. |  | IWG discussion | no | Outcome |
| Cognitive appraisal | Zest for life |  |  | Rapid review | no | Outcome or emotional state |
| Social competence | Group and teamwork capacity | (Team effectiveness) depends on members’ ability to work together in ways that meet task demands, maintain the group, and support individual well-being. |  | Rapid review | no | Not an individual-level attribute |
|  | Adherence to regulations/rules |  |  | Rapid review | no | Behaviour |
|  | Apply knowledge, apply expertise, competency |  |  | Rapid review | no | Overlaps with cognitive capacity |
|  | Attention |  |  | Rapid review | no | Overlaps with cognitive capacity |
|  | Bodily/spatial self-location |  |  | Rapid review | no | Locomotor capacity |
|  | Cognitive resources/functioning |  |  | Rapid review | no | Overlaps with cognitive capacity |
|  | Commitment |  |  | Rapid review | no | Behaviour |
|  | Communication | A process in which individuals use symbols to create and interpret meaning in social interaction; The transmission of information, which may be by verbal (oral or written) or nonverbal means. Humans communicate to relate and exchange ideas, knowledge, feelings, and experiences and for many other interpersonal and social purposes (APA). |  | Rapid review | no | Behaviour |
|  | Consideration |  |  | Rapid review | no | Behaviour |
|  | Decision making ability | Decision making: the cognitive process of choosing between two or more alternatives, ranging from the relatively clear cut (e.g., ordering a meal at a restaurant) to the complex (e.g., selecting a mate). Psychologists have adopted two converging strategies to understand decision making: (a) statistical analysis of multiple decisions involving complex tasks and (b) experimental manipulation of simple decisions, looking at the elements that recur within these decisions (APA). |  | Rapid review | no | Overlaps with cognitive capacity |
|  | Dyadic (familiar/intimate) relationships; contact with others |  |  | Rapid review | no | External, not individual characteristic |
|  | Eudemonic wellbeing |  |  | Rapid review | no | Outcome |
|  | Evaluative (subjective) wellbeing |  |  | Rapid review | no | Outcome |
|  | Hedonic wellbeing |  |  | Rapid review | no | Outcome |
|  | Historical/temporal self-location |  |  | Rapid review | no | Locomotor capacity |
|  | Imagination |  |  | Rapid review | no | Overlaps with cognitive capacity |
|  | impulse, sexual | Impulse control refers to the ability to resist or delay an impulse, drive, or temptation to act.  (impulse control) the ability to resist an impulse, desire, or temptation and to regulate its translation into action (APA).  Sexual self-regulation refers to the processes by which sexual thoughts, feelings, and behaviours are controlled or modified in accordance with personal goals, values, and social constraints. |  | Rapid review | no | Behavioural tendency, encompassed by ‘self-regulation |
|  | Interpersonal connectedness | Social connectedness is an enduring and subjective sense of belongingness and relatedness to others; 1.The connections and interactions, especially ones that are socially and emotionally significant, between two or more people. 2. The pattern or patterns observable in an individual’s dealings with other people. |  | IWG discussion | no | Outcome |
|  | Judgment | The capacity to recognise relationships, draw conclusions from evidence, and make critical evaluations of events and people. |  | Rapid review | no | Overlaps with cognitive capacity |
|  | Language |  |  | Rapid review | no | Overlaps with cognitive capacity |
|  | Memory, retain information and knowledge |  |  | Rapid review | no | Overlaps with cognitive capacity |
|  | Mentalizing | The capacity to understand behavior in terms of underlying mental states, such as thoughts, feelings, intentions, and desires; the ability to perceive and interpret human behaviour in terms of intentional mental states. |  | Rapid review | no | Behaviour |
|  | Mobility |  |  | Rapid review | no | Locomotor capacity |
|  | Perception, capacity to perceive and understand the world, |  |  | Rapid review | no | Overlaps with cognitive capacity |
|  | Physical fitness |  |  | Rapid review | no | Locomotor capacity |
|  | Receive information |  |  | Rapid review | no | Overlaps with cognitive capacity |
|  | Reciprocity |  |  | Rapid review | no | Not a capacity |
|  | Recognition of opportunities or affordances |  |  | Rapid review | no | Overlaps with cognitive capacity |
|  | Reliance |  |  | Rapid review | no | Not a capacity |
|  | Self-care; care for oneself |  |  | Rapid review | no | Behaviour |
|  | Social interaction | any process that involves reciprocal stimulation or response between two or more individuals. These can range from the first encounters between parent and offspring to complex interactions with multiple individuals in adult life. Social interaction includes the development of cooperation and competition, the influence of status and social roles, and the dynamics of group behaviour, leadership, and conformity (APA). |  | Rapid review | no | Behaviour |
|  | Social resilience | The ability of groups or communities to cope with external stresses and disturbances as a result of social, political, and environmental change. |  | IWG discussion | no | Not an individual-level attribute |
|  | Standards and values |  |  | Rapid review | no | Not a capacity |
|  | Structuring and planning tasks; capacity to form projects, reflect and deliberate. | | | Rapid review | no | Overlaps with cognitive capacity |

**Abbreviation:**

IWG: International Working Group

APA: American Psychological Association

**Definitions of the attributes’ Sub-domains:**

1. **Personal agency**: To intentionally make things happen by one’s actions [1].
2. **Cognitive appraisal**: The process of categorising an encounter, and its various facets, with respect to its significance for well-being [2].
3. **Affect:**
   a. Any experience of feeling or emotion, ranging from suffering to elation, from the simplest to the most complex sensations of feeling, and from the most normal to the most pathological emotional reactions. Often described in terms of positive affect or negative affect, both mood and emotion are considered affective states. Along with cognition and conation, affect is one of the three traditionally identified components of the mind [5].
   b. Affect refers to the subjective experience of feeling, encompassing states such as emotions and moods [3].
4. **Adaptability**:
   a. Adaptability refers to an individual’s capacity to adjust their cognitive, behavioural, and emotional responses to effectively manage new, changing, or uncertain situations [4].
   b. 1. The capacity to make appropriate responses to changed or changing situations; 2. the ability to modify or adjust one’s behaviour in meeting different circumstances or different people [5].
5. **Social competence**: effectiveness or skill in interpersonal relations and social situations, increasingly considered an important component of mental health. Social competence involves the ability to evaluate social situations and determine what is expected or required; to recognise the feelings and intentions of others; and to select social behaviours that are most appropriate for that given context. It is important to note, however, that what is required and appropriate for effective social functioning is likely to vary across settings [5].

Note that many attributes can be categorised to more than one sub-domain.

**References**

1. Bandura A. Social cognitive theory: An agentic perspective. *Ann Rev Psychol* 2001;52(1):1-26.

2. Lazarus RS, Folkman S. Stress, appraisal, and coping: Springer publishing company 1984.

3. Scherer KR. What are emotions? And how can they be measured? *Social science information* 2005;44(4):695-729.

4. Martin AJ, Nejad H, Colmar S, Liem GAD. Adaptability: Conceptual and empirical perspectives on responses to change, novelty and uncertainty. *Australian Journal of Guidance and Counselling* 2012;22(1):58-81.

5. American Psychological Association. APA Dictionary of Psychology social competence  [Available from: <https://dictionary.apa.org/social-competence17> December 2025.
